# Toothpastes with Enzymes Support Gum Health and Reduce Plaque Formation

**DOI:** 10.3390/ijerph18020835

**Published:** 2021-01-19

**Authors:** Pune N. Paqué, Patrick R. Schmidlin, Daniel B. Wiedemeier, Florian J. Wegehaupt, Phoebe D. Burrer, Philipp Körner, Shengjile Deari, Michel-Angelo Sciotti, Thomas Attin

**Affiliations:** 1 Clinic of Conservative and Preventive Dentistry, Center of Dental Medicine, University of Zurich, Plattenstrasse 11, 8032 Zurich, Switzerland; patrick.schmidlin@zzm.uzh.ch (P.R.S.); florian.wegehaupt@zzm.uzh.ch (F.J.W.); phoebe.burrer@zzm.uzh.ch (P.D.B.); philipp.koerner@zzm.uzh.ch (P.K.); shengjile.deari@zzm.uzh.ch (S.D.); thomas.attin@zzm.uzh.ch (T.A.); 2 Statistical Services, Center of Dental Medicine, University of Zurich, Plattenstrasse 11, 8032 Zurich, Switzerland; daniel.wiedemeier@zzm.uzh.ch; 3 School of Life Sciences, Institute for Chemistry and Bioanalytics, University of Applied Sciences Northern Switzerland, Hofackerstrasse 30, 4132 Muttenz, Switzerland; michelangelo.sciotti@fhnw.ch

**Keywords:** gingiva, toothbrushing, dentifrice, prophylaxis, prevention, bacteria, gingivitis, low responder, high responder

## Abstract

Enzymes in toothpastes can support host immune responses, and thus maintain oral health. This study aimed to investigate gingival health and the plaque-reducing effects of enzyme-containing toothpastes. A laboratory study tested the antimicrobial potential of different enzyme-containing toothpaste formulations. Two promising formulations (enzyme-containing toothpastes with glucose oxidase and D-glucose with (C+) and without Citrox (C−) Citrox) were investigated in a clinical crossover trial (two slurries: sodium lauryl sulfate-containing (SLS), a toothpaste without SLS (reference), and water). Subjects (*n* = 20) abstained from toothbrushing for four days and rinsed with a toothpaste slurry. Bleeding on probing (BOP) and plaque indices (PI) were measured. A mixed linear model was used to statistically compare the slurries with respect to BOP and PI change. The in vitro bacterial growth-inhibiting evaluation showed the best results for SLS, followed by C+ and C−. The change in BOP and PI exhibited statistically significant differences to water rinsing (BOP; PI changes in % points (difference of the baseline and post-rinse values: water = 8.8%; 90.0%; C+ = −1.4%; 80.4%; SLS = 1.5%; 72.1%; reference = 0.8%; 77.5%; C− = −1.8%; 75.1%). All slurries exhibited anti-gingivitis and anti-plaque effects, resulting in a prophylactic benefit for limited-access regions during brushing.

## 1. Introduction

The presence of oral microorganisms and their potential pathological causes were already described in the 17th century by Antoni van Leeuwenhoek’s microscopic investigations [1]. Attempts to eliminate oral pathogens in the oral cavity by antibiotics and mouth rinses were the consequential implications, and studies investigating more specific anti-biofilm therapies followed [2,3]. Nowadays, most attempts to eliminate oral pathogens are replaced by more biological approaches to promote oral health. The maintenance of a healthy balanced microbiome is hereby the primary goal to prevent oral infections, such as caries and periodontitis [4,5]. General daily oral hygiene regimens by mechanical plaque removal and the application of adequate toothpastes are the main focuses of oral health to prevent and reduce gingivitis [6]. Persisting dental plaque near the gingival sulcus can initiate gingivitis and trigger the progression of oral diseases [7,8]. The most prominent oral diseases, such as caries and periodontitis, develop upon interrupted homeostasis, when dysbiosis occurs [9]. Plaque accumulation induces shifts in the composition of disease-associated microorganisms in the oral cavity and disease can develop as consequence to the elevated pathogen level and host’s immune response. The earliest defense mechanisms against pathogens are based on the nonspecific immune response. The specific immune response, in contrast, comprises the antigen-specific and target-oriented defense by cytokines and inflammatory mediators [10]. As preventive measure, plaque control can re-establish healthy gingival conditions and is effective against gingivitis [7]. In this context, new toothpaste formulations are constantly developed and aim to support a biological approach and contain antibacterial agents, enzymes, or proteins to even boost the defense mechanisms of saliva mainly against pathogenic oral pathogens if possible.

One important nonspecific immune response in saliva is based on the lactoperoxidase (LPO) system. It renders the natural saliva defense by oxidizing salivary thiocyanate (SCN−) in the presence of elevated hydrogen peroxide levels. Hydrogen peroxide and SCN− exhibit antimicrobial activities and have already been shown to inhibit the growth of cariogenic bacteria in vitro [11,12,13]. In the oral cavity, hydrogen peroxide is either secreted by the salivary glands or produced by several bacterial species [13]. Enzyme-containing toothpastes with an implemented LPO system frequently depend on the enzyme glucose oxidase (GOX) for the production of hydrogen peroxide and SCN− for the peroxide-dependent production of antibacterial hypothiocyanite by LPO [14]. The efficacy of enzyme-containing toothpastes has also been exhibited in clinical studies [15], i.e., in a meta-taxonomic study, which indicates bacterial shifts toward an overall healthier oral microbiome after toothbrushing with respective enzyme-containing toothpastes [16]. Toothpaste formulations with implemented enzymes and proteins must comply with certain requirements, such as the locking of the enzymatic cascade during toothpaste production and tube storage, ensuring that enzyme activation is triggered only during toothbrushing, and the formulation of the stable amounts of enzymes to exhibit measurable oral health benefits. These benefits should ideally be investigated separately from the mechanical plaque removal capacities of toothpastes to avoid effects of interindividual brushing habits and different mechanical cleaning efficacies of toothpastes (due to their abrasive load). Separating chemical actions of toothpastes from the mechanical effects during toothbrushing requires appropriate approaches. Addy and his colleagues described a method to analyze the plaque-reducing effects of toothpaste slurries during a four-day period without bristle contact [17]. This method is also suitable for the analysis of gingival health and the plaque reducing effects of toothpastes without considering their mechanical cleaning potentials. Detecting the chemical effects of toothpaste slurries on gums and plaques solely requires strong controls to keep up with. Effective plaque control and gingivitis-reducing effects are shown for sodium lauryl sulfate (SLS)- and triclosan-containing toothpastes [18,19,20]. However, the anionic surfactant SLS is assumed to cause desquamative effects on oral mucosa [21,22]. Additionally, toothpaste formulations without SLS are exhibited to reduce recurrent aphthous ulcers (RAU) compared with SLS-containing formulations [23]. Triclosan supplements can protect these potential side effects of SLS by reducing the increasing mucosal permeability of oral mucosa and reducing RAU incidence [24]. This triclosan/SLS adjustment of side effects, nonetheless, depends on the relative amount of triclosan and SLS in toothpaste formulations, and protection against its side effects is not always offered [25,26].

Hence, the following study aimed to (1) prior screen different SLS-free enzyme-containing toothpaste prototypes in vitro using a simple agar disc diffusion assay with an exemplary caries-associated bacterial strain (*Streptococcus mutans*) and (2) to clinically assess the related gingivitis- and plaque-reducing effects of the most promising two test enzyme-containing toothpastes in comparison with SLS-containing toothpastes, a reference toothpaste without enzymes or SLS, and water rinsing using a four-day plaque model with a novel splint approach. The null hypothesis assumed that enzyme-containing toothpastes without SLS and triclosan show the same gingivitis- and plaque-reducing effects than water (control) and SLS- and triclosan-containing toothpastes.

## 2. Materials and Methods

This two-part study consisted of a preliminary laboratory test to evaluate the antibacterial efficacies of divergent prototype toothpastes with enzymatic activity and a randomized controlled trial (RCT), in which the most relevant and potentially most active prototypes were tested in vivo. The RCT was approved by the local ethics committee (BASEC-no. 2016-00266) and was in accordance with the principles of the Declaration of Helsinki. Moreover, the trial was registered in the Internet Portal of the German Clinical Trials Register (DRKS00009823) and the Swiss National Clinical Trials Portal (SNTP000001645). A written informed consent was obtained from all the participants. The study complied with the Consort 2010 checklist of information to include when reporting a randomized trial (Supplemented Table S1).

### 2.1. Laboratory Study

#### 2.1.1. Toothpaste Slurries

The prototype toothpastes were prepared to exhibit enzymatic activity upon exposure to oxygen. Table 1 lists the compositions of the base toothpaste formulation. Active ingredients, such as glucose oxidase (GOX), D-glucose, vitamin C, sodium bisulfite, and antibacterial adjuncts, such as Citrox [27], were added in distinct concentrations (Table 2). The base formulation comprised commercially available Enzycal 950 toothpaste (CURADEN, Kriens, Switzerland; Supplemented Table S2). The enzymatic activity that was strived for was based on the atmospheric oxygen-triggered conversion of D-glucose to D-glucono-1.5-lactone and hydrogen peroxide. The divergent prototypes, which were tested in the preliminary study, were also tested for the long-term stability of the enzymatic activity (data not published).

Furthermore, four toothpastes were clinically tested under blinded conditions using William’s square design to randomly allocate sequences to the subjects: two toothpastes, which exhibited the highest antibacterial efficacy in a pilot agar diffusion test, one reference, consisting of the same formulation without enzymatic activity, and a commercially available reference toothpaste (Table 1). All toothpastes were applied in identical 15 mL tubes, which were double-coded with colors and letters. The codes for the sequences (letters) and colors were sealed in an envelope and decoding was only possible after the statistical analysis.

**Figure 1:**
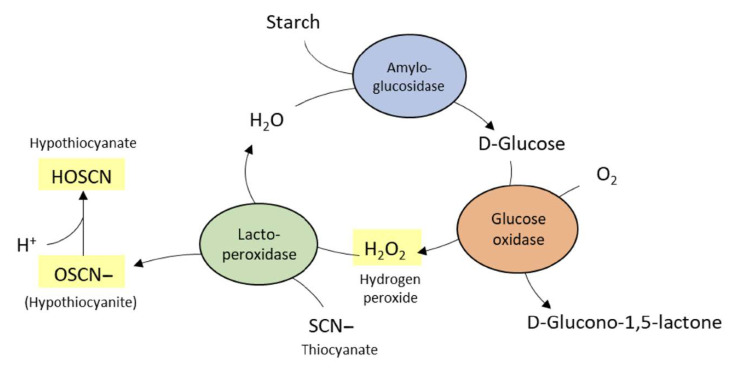
The peroxidase system of the natural saliva defense system generates hydrogen peroxide by the enzyme glucose oxidase and initiates the oxidation of thiocyanate (SCN−) to hypothiocyanite (HOSCN/OSCN−) by the enzyme lactoperoxidase (LPO). The enzyme-containing toothpastes abstain from starch and amyloglucosidase for D-glucose delivery. D-Glucose was directly formulated in the toothpastes together with glucose oxidase. Thiocyanate and LPO are only added to the prototype 755 (see Table 2). The prototypes 255, 258, and C58 rely on the endogenous salivary LPO and thiocyanate for the production of hypothiocyanite.

The enzymatic activity of the enzyme-containing toothpastes was initiated upon oxygen exposure. To ensure the longevity of the enzymatic activity, the subjects were instructed closely to generate a foamy slurry by brushing the oral rippled splint with the respective toothpaste and closing all tubes correctly after usage. Figure 1 describes the background of the enzymatic activity.

#### 2.1.2. Laboratory Testing

The different prototypes of toothpastes with varying compositions were first investigated in a laboratory setting. A simple agar disc diffusion assay was employed to measure the antibacterial efficacies of toothpaste slurries by means of measuring the zones of inhibition. For this purpose, a pure culture of *S. mutans* (OMZ 918) was also utilized. The respective colonies were gained from Colombia sheep’s blood agar plates (bioMérieux, Marcy l’Etoile, France) and propagated planktonic in a mixture of a 30% saliva solution and a 70% modified fluid universal medium (mFUM) [28,29,30]. The whole saliva from one healthy subject was centrifuged (2 × 30 min, 13,400 rpm), and the supernatant was diluted 1:2 in 0.9% sodium chloride prior to sterile filtration (TPP syringe filters 0.2 µm pores, Faust, Schaffhausen, Switzerland). After 24 h of anaerobic incubation (GENbox anaer and GENbag anaer, bioMérieux), the *S. mutans* broth culture was adjusted to an optical density (OD_550_) of 1 and used to streak the mFUM agar plates (mFUM with 1% Agar nobile Beckton Dickinson, Allschwil, Switzerland). Additionally, the sterile filter paper disks with a diameter of 9 mm (Gel-Blotting Paper, Whatman^TM^, Fisher Scientific Sa, Wohlen, Switzerland) were applied on each plate and immediately covered with 100 µL of test solutions. A total of 0.2% chlorhexidine (Chlorhexamed, GlaxoSmithKline Consumer Healthcare GmbH & Co. KG, München, Germany), 0.1% and 0.05% H_2_O_2_ solutions, and supernatant of a commercially available toothpaste (Colgate Total^®^ Original, Colgate-Palmolive Company, Therwil, Switzerland) with strong antibacterial efficacy were used as controls on each plate. The supernatants of prototype toothpaste slurries and toothpastes without antibacterial activity (negative control) were tested in triplets. Table 2 summarizes the respective evaluations of this study.

### 2.2. Clinical Study

#### 2.2.1. Study Population

To detect differences in plaque- and gingivitis-reducing effects, mainly high responders with respect to gingivitis formation in accordance with and modified from Trombelli and his colleagues were included in the clinical study [7,8]. The inclusion criteria were a caries-free dentition with a minimum number of posterior teeth (at least three neighboring side teeth) without restorations to avoid plaque retentive niches and surfaces, which might hamper the disclosing and cleaning, respectively.

**Figure 2:**
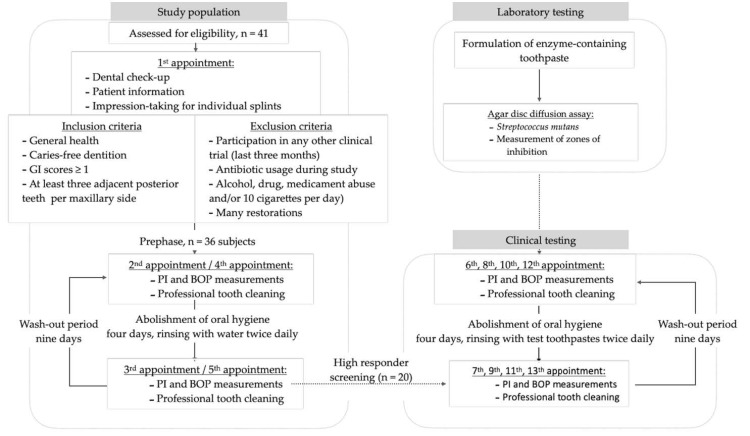
Flow chart with details on subject enrollment and intervention in this study. Section 2.2 (Section 2.2.1 & Section 2.2.2) Study Design.

Systemically healthy subjects (minimum age of 18 years, detailed inclusion criteria in Figure 2) were recruited in the first evaluation phase, which included two runs of each four-day abstinence from oral hygiene procedures with pure water rinsing twice a day. Subjects with a decline of bleeding on probing (BOP) scores after four days of omitted toothbrushing were excluded from further tests. Only subjects showing equal or increasing BOP scores were assessed as high responders and subjected to the test phase. Participating subjects were instructed to maintain their regular diet and do not rinse their teeth during the study with mouth rinses or use antiseptics.

The recruitment of subjects was performed from July to November 2016 at the Center for Dental Medicine, Zurich, Switzerland, disseminating information on the website and flyers at local university facilities. Forty-one healthy subjects were interested in participating and were initially screened for the inclusion/exclusion criteria (Figure 2). Twenty healthy volunteers aged 19–55 (mean: 29.6 years; stratified for gender, 10 males) were enrolled in the trial, starting with interventions from August until March 2017 (Figure 2).

**Figure 3:**
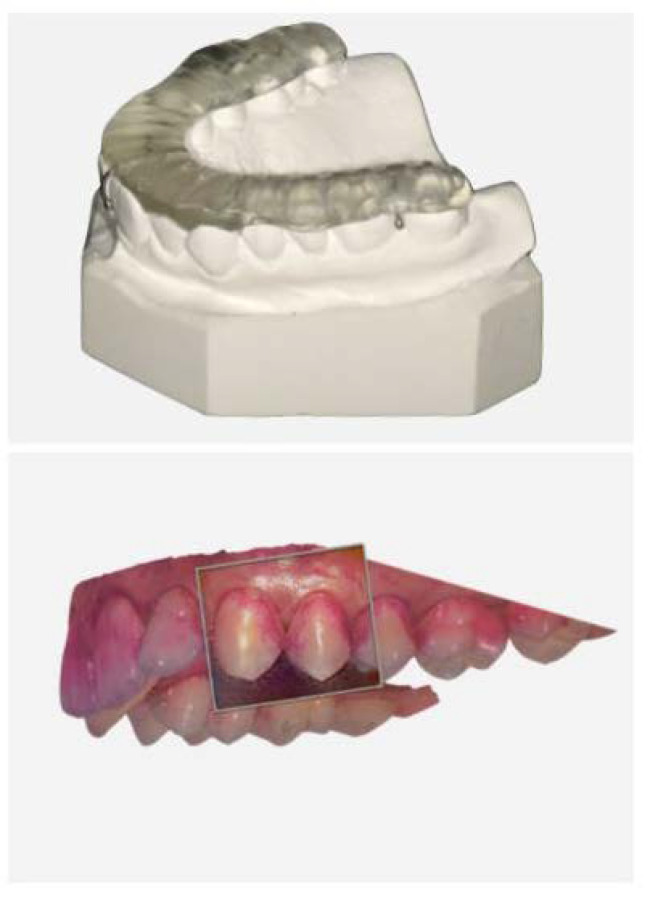
Representative oral splint applied during brushing to produce the foamy slurry of the test toothpastes and representative oral scan of the upper jaw (3Shape Trios, TRC; 3Shape, Copenhagen, Denmark) after four-day oral hygiene abstinence. The subjects were instructed to brush the rippled splint with the test toothpastes to avoid the actual mechanical intervention of the teeth.

The study was a randomized controlled, stratified, crossover study at one center (Center for Dental Medicine, University of Zurich, Switzerland). All the subjects attended either five or 13 appointments. All the subjects were informed about the study details and screened for potential inclusion and exclusion criteria at the first visit. In addition, an alginate impression was taken from the upper jaw to produce individual splints (Figure 2), which served as “scrub-rail” during the test phase (details below). A dental technician prepared the splints for each study subject. The participants who complied with the inclusion criteria started the evaluation phase, which consisted of two runs of water rinsing. At the second and fourth visits, plaque accumulation was disclosed with a dye (paro^®^ plak, Esro AG, Kilchberg, Switzerland) to document plaque accumulation [31]. Bleeding on probing (BOP) [32] was tested using standardized probes (Click-Probe^®^, KerrHawe SA, Bioggio, Switzerland). Subsequently, the subjects received professional tooth cleaning and were asked to abolish oral hygiene for four days and were advised to only rinse with tap water twice a day. On the third and fifth visits, BOP and plaque indices (PI) were re-documented prior to the professional tooth cleaning to complete the water runs of the evaluation phase. After each run, a wash-out period of nine days was interposed (Figure 2). After the evaluation phase, the collected data, i.e., BOP and PI, were evaluated, and those who revealed equal or increasing BOP scores were engaged as high responders to attend the actual test phase, which comprised four runs with toothpaste slurries. At the beginning of each test run, the BOP and PI scores were collected, and a professional tooth cleaning was performed using ultrasonic devices and rubber cups. Thereafter, high responders received their individual splint, a standardized toothbrush (Paro M43, Esro AG), a timer, a syringe, and a test toothpaste (either an enzyme-containing test toothpaste without sodium lauryl sulfate-containing glucose oxidase enzymes, D-glucose, with (C+) or without Citrox (C−), or an enzyme-free reference toothpaste consisting of SLS- and triclosan-free toothpastes (reference) or an SLS- and triclosan-containing toothpaste (SLS)) with instructions. Again, the subjects were asked to abolish all the oral hygiene procedures for four days and rinse twice daily with toothpaste slurries generated with the rippled splints as follows: the subjects were asked to insert the splint (Figure 3), apply the toothpaste to the toothbrush, apply 2 mL tap water to the mouth using the syringe, and start brushing the splint for 30 s to generate a toothpaste slurry without actually brushing the teeth. Afterward, the splint was removed, and the slurry rinsing continued for 90 s. This procedure was repeated twice daily for four days until the next BOP/PI collection and tooth cleaning with the subsequent wash-out phase.

All the test toothpastes were handed out in identical tubes marked with letters and colors. The corresponding code affiliations were sealed in a letter until the end of the study and were opened after the statistical analysis. Each subject tested all the test toothpastes in a controlled order (see Statistical Analysis).

#### 2.2.2. Clinical Parameters

All the examinations were performed by one trained and blinded dentist (PNP). Cleaning efficacy [31] was recorded at the baseline and after rinsing with the respective slurries. The BOP [32] was measured at baseline and after four days of rinsing. Differences in the BOP of both timepoints (post-rinsing BOP minus pre-rinsing BOP) were employed for analysis. A calibration to determine the outcome of the cleaning efficacy and the BOP was not performed because red-colored plaque and blood, respectively, ensured a clear differentiation to determine both parameters. In addition, the documentation and calculation of the percentage of clinical outcome data were double-checked by the dental assistant and the trained dentist on the same day of the examinations.

### 2.3. Statistical Analysis

The different test toothpastes were distributed to the subjects according to a William’s square design to reduce the first-order carry-over effects. Descriptive statistics were calculated for both endpoints BOP and PI changes (mean, standard deviation (SD), median and interquartile ranges).

The two mixed linear models were employed to detect the differences between toothpaste slurries with regard to the BOP and PI changes, respectively, during the rinsing runs. The changes in BOP and PI were both expressed as percentage points, and each was modeled as a function of the fixed factor toothpaste slurry (with five levels, including the water rinses) and a patient as a random factor (with 20 levels). The pairwise comparisons between the toothpastes were then performed on their estimated marginal means, and the resulting *p*-values were adjusted for multiple testing according to Tukey’s test. The estimated marginal means and their standard errors (estimated mean (% point change) ± SE (% point change)) are reported throughout the manuscript. Method for comparing a family of five estimates (*p*-value adjustment). The significance level was set to α = 0.05, and all the calculations were performed with the statistical software R [33] using the following packages [34,35].

## 3. Results

### 3.1. Laboratory Testing

Out of the six tested prototype toothpastes, three compositions exhibited higher antibacterial efficacies against *S. mutans* in an agar diffusion assay (Supplemented Data S1). Two prototypes (258 and C58) showed the largest zones of inhibition in the agar assay (Table 2), both consisting of GOX and D-glucose with sodium bisulfate. C58 was additionally implemented with Citrox, and the SLS- and triclosan-containing reference toothpastes showed, nevertheless, the overall strongest antibacterial efficacy against *S. mutans* with respective inhibition zones of 46.8 mm ± 2.2 mm (Table 2).

### 3.2. Clinical Outcomes

**Figure 4:**
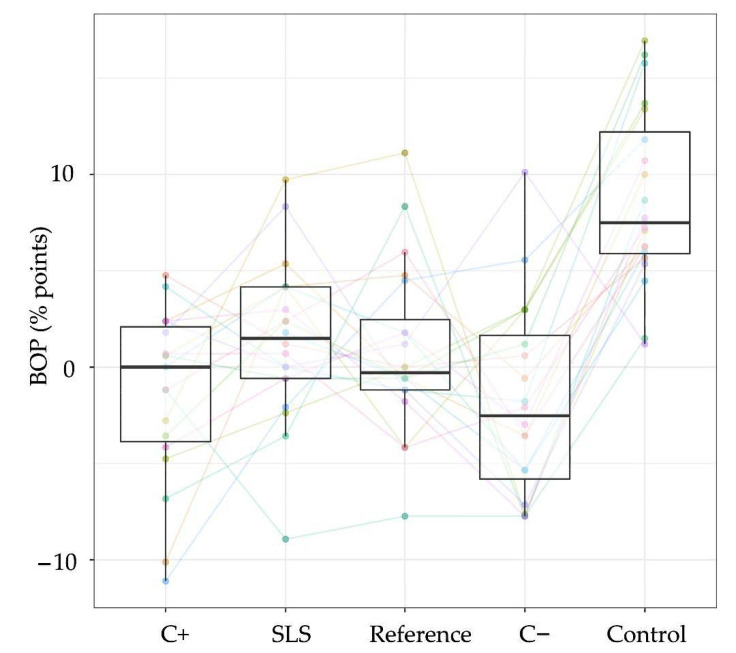
The boxplots of differences in bleeding on probing (BOP) after rinsing and at the baseline of each test toothpaste and the water application are presented for all the subjects (*n* = 20, colored), showing median percentages, 25th and 75th quartiles, standard deviation, and outliers. The data of both water runs, which were performed prior to the test phase, were averaged. C+ = enzyme-containing toothpaste with Citrox; SLS = SLS-containing reference toothpaste; placebo = reference toothpaste without SLS and without enzymes; C− = enzyme-containing toothpaste without Citrox; control = mean values after water rinsing.

**Figure 5:**
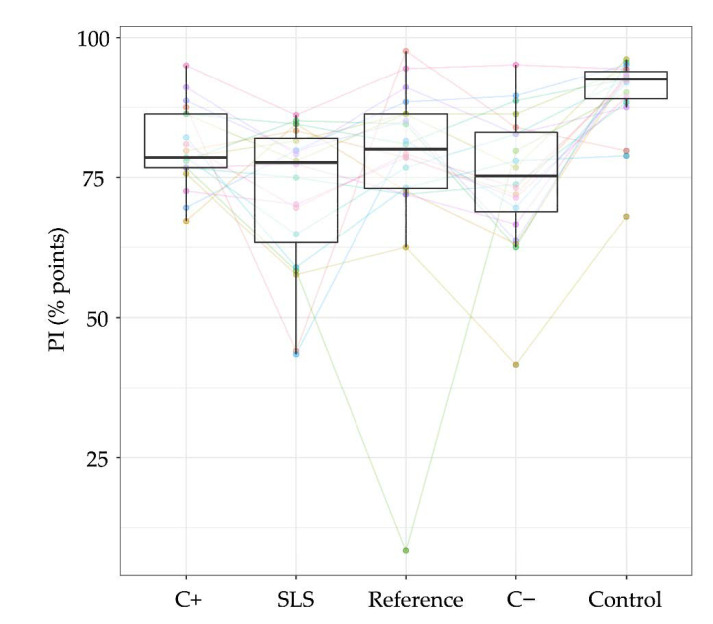
Boxplots of estimated means (plaque indices) of each test toothpaste and the water application of all the subjects (*n* = 20, colored) in percentage (%), showing median percentages, 25th and 75th quartiles, standard deviation, and outliers. The plaque indices of both water runs, which were performed prior to the test phase, were averaged. C+ = enzyme-containing toothpaste with Citrox; SLS = SLS-containing reference toothpaste; placebo = reference toothpaste without SLS and without enzymes; C− = enzyme-containing toothpaste without Citrox; control = mean values after water rinsing.

Four slurries were analyzed for their ability to reduce plaque formation without mechanical intervention and their effect on BOP. All the subjects utilized water rinses at the beginning, which manifested the best outcome in terms of plaque formation (mean ± SD: 90% points ± 2.8% points) and an increase in BOP (8.8% points ± 1.0% points) compared with the test slurries. The SLS-containing reference toothpaste showed the highest plaque control by pure rinsing (72.1% points ± 2.8% points) followed by C− (75.1% points ± 2.8% points). C+ and the placebo showed similar outcomes (C+: 80.2% points ± 2.8% points; placebo: 77.5% points ± 2.8% points). In terms of BOP, the best results were obtained using both enzyme-containing toothpastes (C+: −1.4% points ± 1% point; C−: −1.9% points ± 1% point), compared with the reference toothpastes (SLS: 1.5% points ± 1% point; placebo: 0.8% points ± 1% point). Statistically, all four toothpaste slurries showed significant differences to rinsing with water with respect to BOP (all test groups: *p* < 0.0001) and plaque (C+: *p* = 0.05; Colgate Total Original: *p* < 0.0001; placebo: *p* = 0.05; C−: *p* = 0.05; Table 3). No statistically significant differences could be detected within the four toothpaste slurries, neither for BOP nor for PI (Figure 4 and Figure 5).

## 4. Discussion

This study evaluated the efficacy of divergent SLS-free enzyme-containing toothpaste formulations in laboratory testing. The potentially most active prototypes were further tested to assess the related gingivitis- and plaque-reducing effects in comparison with SLS-containing toothpastes, a placebo toothpaste without enzymes, and water rinsing using a four-day plaque model. The main findings of the laboratory testing showed superior antimicrobial effects using the SLS- and triclosan-containing toothpaste compared with all other groups. This strong effect against *S. mutans* generated large zones of inhibition, namely, 46.8 mm ± 2.2 mm (Table 2), which was in line with other studies [20] and amounted for more than twice the diameter of all enzyme-containing toothpastes in the agar disc diffusion assay. Nonetheless, this superior effect of the SLS- and triclosan-containing toothpaste was leveled with the other toothpastes in the four-day rinsing phase to less pronounced effects in plaque accumulation and BOP. Significant differences in BOP and plaque formation were shown for all toothpastes compared with water rinsing. However, only little differences within all four toothpaste groups were detectable in this study: the enzyme-containing test toothpastes C+ and C− resulted in both reduced BOP values compared with the placebo and SLS reference and SLS groups. The null hypothesis was, therefore, partially rejected. The SLS and C− groups showed the highest reduction in plaque formation (Table 3). Colgate, which served as SLS- and triclosan-containing test toothpaste, resulted in expectedly strong gingivitis- and plaque-reducing effects; however, both enzyme-containing test toothpastes were able to keep up with it, exhibiting comparable effects on gum health and plaque formation. The effective plaque control and gingivitis-reducing effects of the control toothpaste Colgate were described before and confirmed in the current laboratory and clinical experiments [18,19,20]. The multi-factorial mode of action was described as an interplay between triclosan, a co-polymer and zinc citrate, which complement one another, leading to the inhibition of glycolysis, bacterial proteases, as well inhibition of the interleukin-induced prostaglandin E_2_ production and overall reduction of Gram-positive and Gram-negative bacteria [36,37]. The enzyme-containing toothpastes, however, were able to unfold their antibacterial effects more distinctively during the clinical experiments. The antimicrobial efficacy was based on the atmospheric oxygen-triggered conversion of D-glucose to D-glucono-1.5-lactone and hydrogen peroxide. This was achieved during the experiments, when participants brushed the splint to produce and activate the slurry with atmospheric oxygen. Surprisingly, however, more pronounced effects were measured in the clinical experiments than on the single species level during the laboratory testing. Possible reasons might include the activation and incubation time of the toothpastes during the diffusion assays. The slurries were mixed and activated by vortexing, and applied on the disc diffusion assay. The contact time with direct atmospheric oxygen might have been shorter, than the clinical brushing time of 2 min, since the agar discs were incubated anaerobically and further conversion to hydrogen peroxide might have been reduced. Therefore, the in vitro antibacterial effects may be primarily based on antibacterial actions of the supplements, such as Citrox and other ingredients rather than their combined action with the enzymes, which led to the overall reduced antibacterial efficacies in the laboratory testing compared to the clinical experiments. The antibacterial adjuncts used contain many bioflavonoids, which were studied in laboratory experiments with different oral bacteria and Candida species [27,38]. It is derived from citrus fruits and working concentrations of 1–2%v/v were recommended to effectively inhibit the bacterial growth in biofilms [27]. Recently, recommendations were published to use oxidizing agents such as Citrox as supplement in mouth rinses to prevent Covid-19 infections and progression [39], next to other prevention protocols and the appropriate patient management to reduce the risk of infection [40]. In this context, the authors highlighted the need of clinical trials to evaluate the properties of the bioflavonoids further.

In addition to the antibacterial effects of the implemented enzymes and antibacterial adjuncts, some bacterial growth-inhibiting effects might also be based on the high glycerin [41] and sorbitol amount [42] in the toothpastes (Supplemented Table S2). We observed, that these supplements unfold their antibacterial properties not on agar plates (Table 2, Supplemented Data S1), but apparently under in vivo conditions. This might at least explain the lack of measurable differences between the reference used, and the enzyme-containing toothpastes in the clinical evaluation (Figure 4 and Figure 5).

The laboratory study was designed to screen the antibacterial properties of the different prototypes on *S. mutans*. Regarding the anti-gingivitis effects of the toothpastes; however, the testing would also have been interesting with gingivitis- or periodontitis-associated species. *S. mutans* was used to simplify comparisons with other studies, which most often apply this species in similar diffusion assays [20,43,44,45].

Addy et al., who described the applied clinical study protocol to analyze rinses during a four-day period, did not observe any plaque-reducing effects by the use of different toothpastes after 96 h compared with rinsing with chlorhexidine [17]. Unfortunately, besides the information on fluoride contents in the applied toothpastes, only little details were communicated in the mentioned study to enable deeper comparisons. The lack of significant differences within the toothpastes employed in this study exhibited equal anti-plaque and anti-gingivitis efficacies between the test groups. Notwithstanding, there appeared to be a high heterogeneity between the study subjects, and the change in BOP indicated high heterogeneity regarding the toothpaste effects between the subjects (Figure 4). The measured plaque scores after 96 h, however, showed for most study subjects very similar tendencies for each toothpaste (Figure 5). Some host responses, such as plaque-induced gingivitis, are known to vary significantly between individuals. For instance, in healthy subjects, plaque accumulation leads to divergent clinical parameters, such as plaque index, gingival index, or gingival crevicular fluid volume [8], showing that healthy subjects react differently to plaque accumulation over time. These clinical differences were classified as the periodontal-resistant and periodontal-insufficient groups [46] or as high and low responders, respectively [8]. The response of both groups to gingivitis therapy (toothbrushing with a fluoride toothpaste) reestablished healthy gums for high and low responders similarly [7]. In this study, a modified version of high and low responder discrimination was applied to reduce heterogeneity in study subjects. The pre-phase, which initially consisted of 36 subjects, was utilized to identify subjects, which exhibited high responder characteristics. The plaque response during pre-phase was, however, only observed during a four-day period twice (instead of three weeks as described above [8]).

The actual sample size of 20 subjects was mainly chosen on the basis of previous studies, which investigated anti-plaque or anti-gingivitis effects after rinsing [17,47,48,49,50]. The sample size turned out to be sufficient due to a calculated post hoc power of 99.9% (PI: water vs. toothpaste rinses). A higher number of subjects might have balanced the differences and revealed more statistical differences between some of the toothpastes. However, all the test toothpastes revealed the plaque scores of ranges between 70% and 80% already after 96 h of plaque accumulation. The BOP was slightly reduced after rinsing with enzyme-containing toothpastes and prolonged using the reference toothpastes. It seems rather questionable if differences in this range cause clinically discriminable or clinically relevant oral health conditions. A longer observation period, however, would be interesting to differentiate between the toothpastes.

On the one hand, this suggests that new enzyme-containing toothpastes can exhibit comparable degrees of anti-plaque and anti-gingivitis effects compared with formulations with SLS or triclosan. On the other hand, it points out the need for the long-term investigations and analyses of different enzyme-containing toothpaste formulations. For instance, Midda et al. implied oral health benefits in subjects using enzyme-containing toothpastes after three months [15].

## 5. Conclusions

Rinsing with toothpaste slurries to evaluate the in vivo efficacy of toothpaste slurries seems a promising tool to screen anti-plaque and anti-gingivitis effects during short observation periods without mechanical brushing using a rippled splint. All tested slurries exhibited anti-gingivitis and anti-plaque effects, resulting in a prophylactic benefit for less accessible regions during brushing.

## Figures and Tables

**Table 1:** Composition of six prototype toothpastes used in the in vitro preliminary testing. The prototypes in bold (258, C58) were applied as enzymatic-containing toothpastes in the randomized clinical trial.

Prototype	Enzymes (%)	D-Glucose (%)	Vitamin C (%)	Sodium Bisulfite (%)	SCN− (%)	Citrox (%)
000	0	0	0	0	0	0
255 *	GOX 0.5	1	0.1	0	0	0
755 *	GOX 0.25 LPO 0.25	0.4	0.004	0	0.1	0
258 *	GOX 0.5	1	0	0.2	0	0
C00	0	0	0	0	0	1
C58 *	GOX 0.5	1	0	0.2	0	1

GOX = glucose oxidase; LPO = lactoperoxidase; SCN− = thiocyanate; * prototype manufacturing under oxygen exclusion in glove boxes.

**Table 2:** Mean diameter (mm) in agar diffusion tests with *S. mutans* using ten toothpaste prototypes, hydrogen peroxide solutions (0.1 and 0.05%), and a sodium lauryl sulfate-containing control toothpaste (sodium lauryl sulfate (SLS) = Colgate, reference control toothpaste).

Prototype/ Test Solution	Mean (mm)	SD (%)
000	–	–
255	11.9	0.8
755	–	–
258	20.4	5.4
C00	–	–
C58	19.2	3.9
0.1% H_2_O_2_	16.1	3.3
0.05% H_2_O_2_	14.3	3.0
SLS	46.8	2.2

**Table 3:** Descriptive mean values with standard deviation (SD), median, and interquartile ranges (IQR), as well as estimated mean values and standard error (SE) of all toothpastes and rinsing with water for both bleeding on probing (BOP) and plaque indices (PI) changes (% points; difference of the baseline and post-rinse values). The statistically significant differences in toothpaste slurries to water are marked with stars; * 0.05 > *p* ≥ 0.01, ** 0.01 > *p* ≥ 0.001, *** *p* < 0.001.

	Descriptive Statistics								Estimated Means			
	BOP				PI				BOP		PI	
Rinses	Mean	SD	Median	IQR	Mean	SD	Median	IQR	Mean	SE	Mean	SE
Water	8.8	4.6	7.5	6.3	90.0	6.9	92.6	4.8	8.8	1.0	90.0	2.8
Reference	0.8	4.4	−0.3	3.7	77.5	18.4	80.1	13.2	0.8 ***	1.0	77.5*	2.8
C+	−1.4	4.5	0.0	6.0	80.4	7.2	78.6	9.5	−1.4 ***	1.0	80.2 *	2.8
C−	−1.8	5.0	−2.5	7.4	75.1	12.2	75.3	14.1	−1.9 ***	1.0	75.1 **	2.8
SLS	1.5	4.2	1.5	4.8	72.1	13.3	77.7	18.6	1.5 ***	1.0	72.1 ***	2.8

## Data Availability

The data presented in this study are provided in the Supplemental Table S3.

## Supplementary Materials

The following are available online at https://www.mdpi.com/1660-4601/18/2/835/s1, Data S1: Representative Images of the Agar Disc Diffusion Assays Using Six Prototype Toothpastes on *S. mutans*. The panels show the zones of inhibition around the discs after application of different prototypes and rinses, focusing on (a) the placebo 000 and the enzyme-containing prototype 255, (b) the reference toothpaste Colgate; (c) the enzyme-containing prototypes 755 and 700, (d) enzyme-containing prototype 255, with references of 0.05% and 0.1% hydrogen peroxide, (e) and (f) the enzyme-containing prototypes C58, C00, 268, and other tested prototypes. Table S1: Consort 2010 checklist of information to include when reporting a randomized trial. Table S2: Base Formulation of Enzyme-Containing Toothpastes.

## References

[1] Leeuwenhoek A., Blois A., Verkolje J., Kroonevelt H. (1697). Arcana Naturae Detecta, Philos Trans R Soc Lond.

[2] Black G. (1884). The Formation of Poisons by Micro-Organisms: A Biological Study of the Germ Theory of Disease.

[3] Miller W. (1890). The Micro-Organisms of the Human Mouth.

[4] Hajishengallis G., Darveau R., Curtis M. (2012). The keystone-pathogen hypothesis. Nat. Rev. Microbiol..

[5] Marsh P. (1994). Microbial ecology of dental plaque and its significance in health and disease. Adv. Dent. Res..

[6] Van der Weijden F., Slot D. (2015). Efficacy of homecare regimens for mechanical plaque removal in managing gingivitis a meta review. J. Clin. Periodontol..

[7] Trombelli L., Scapoli C., Orlandini E., Tosi M., Bottega S., Tatakis D. (2004). Modulation of clinical expression of plaque-induced gingivitis. III. Response of “high responders” and “low responders” to therapy. J. Clin. Periodontol..

[8] Trombelli L., Tatakis D., Scapoli C., Bottega S., Orlandini E., Tosi M. (2004). Modulation of clinical expression of plaque-induced gingivitis. II. Identification of “high-responder” and “low-responder” subjects. J. Clin. Periodontol..

[9] Dewhirst F., Chen T., Izard J., Paster B., Tanner A., Yu W., Lakshmanan A., Wade W. (2010). The human oral microbiome. J. Bacteriol..

[10] Lo Giudice G., Nicita F., Militi A., Bertino R., Matarese M., Curro M., Damiano C.S., Mannucci C., Calapai G. (2019). Correlation of s-IgA and IL-6 Salivary with Caries Disease and Oral Hygiene Parameters in Children. Dent. J. (Basel).

[11] Lumikari M., Soukka T., Nurmio S., Tenovuo J. (1991). Inhibition of the growth of Streptococcus mutans, Streptococcus sobrinus and Lactobacillus casei by oral peroxidase systems in human saliva. Arch. Oral Biol..

[12] Ryan C., Kleinberg I. (1995). Bacteria in human mouths involved in the production and utilization of hydrogen peroxide. Arch. Oral Biol..

[13] Thomas E., Milligan T., Joyner R., Jefferson M. (1994). Antibacterial activity of hydrogen peroxide and the lactoperoxidase-hydrogen peroxide-thiocyanate system against oral streptococci. Infect. Immun..

[14] Magacz M., Kędziora K., Sapa J., Krzyściak W. (2019). The significance of lactoperoxidase system in oral health: Application and efficacy in oral hygiene products. Int. J. Mol. Sci..

[15] Midda M., Cooksey M. (1986). Clinical uses of an enzyme-containing dentifrice. J Clin Periodontol.

[16] Adams S., Arnold D., Murphy B., Carroll P., Green A., Smith A., Marsh P., Chen T., Marriott R., Brading M. (2017). A randomised clinical study to determine the effect of a toothpaste containing enzymes and proteins on plaque oral microbiome ecology. Sci. Rep..

[17] Addy M., Willis L., Moran J. (1983). Effect of toothpaste rinses compared with chlorhexidine on plaque formation during a 4-day period. J. Clin. Periodontol..

[18] Davies R., Ellwood R., Davies G. (2004). The effectiveness of a toothpaste containing triclosan and polyvinyl-methyl ether maleic acid copolymer in improving plaque control and gingival health: A systematic review. J. Clin. Periodontol..

[19] Haraszthy V., Zambon J., Sreenivasan P. (2010). Evaluation of the antimicrobial activity of dentifrices on human oral bacteria. J. Clin. Dent..

[20] Randall J., Seow W., Walsh L. (2015). Antibacterial activity of fluoride compounds and herbal toothpastes on Streptococcus mutans: An in vitro study. Aust. Dent. J..

[21] Green A., Crichard S., Ling-Mountford N., Milward M., Hubber N., Platten S., Gupta A., Chapple I. (2019). A randomised clinical study comparing the effect of Steareth 30 and SLS containing toothpastes on oral epithelial integrity (desquamation). J. Dent..

[22] Herlofson B., Barkvoll P. (1993). Desquamative effect of sodium lauryl sulfate on oral mucosa. A preliminary study. Acta Odontol. Scand..

[23] Herlofson B., Barkvoll P. (1994). Sodium lauryl sulfate and recurrent aphthous ulcers. A preliminary study. Acta Odontol. Scand..

[24] Healy C., Cruchley A., Thornhill M., Williams D. (2000). The effect of sodium lauryl sulphate, triclosan and zinc on the permeability of normal oral mucosa. Oral Dis..

[25] Baert J., Veys R., Ampe K., De Boever J. (1996). The effect of sodium lauryl sulphate and triclosan on hamster cheek pouch mucosa. Int. J. Exp. Pathol..

[26] Skaare A., Eide G., Herlofson B., Barkvoll P. (1996). The effect of toothpaste containing triclosan on oral mucosal desquamation. A model study. J. Clin. Periodontol..

[27] Hooper S., Lewis M., Wilson M., Williams D. (2011). Antimicrobial activity of Citrox bioflavonoid preparations against oral microorganisms. Br. Dent. J..

[28] Loesche W., Hockett R., Syed S. (1972). The predominant cultivable flora of tooth surface plaque removed from institutionalized subjects. Arch. Oral Biol..

[29] Tawakoli P., Sauer B., Becker K., Buchalla W., Attin T. (2015). Interproximal biofilm removal by intervallic use of a sonic toothbrush compared to an oral irrigation system. BMC Oral Health.

[30] Thurnheer T., Gmur R., Guggenheim B. (2004). Multiplex FISH analysis of a six-species bacterial biofilm. J. Microbiol. Methods.

[31] O’Leary T., Drake R., Naylor J. (1972). The plaque control record. J. Periodontol..

[32] Ainamo J., Bay I. (1975). Problems and proposals for recording gingivitis and plaque. Int. Dent. J..

[33] R Core Team (2018). A Language and Environment for Statistical Computing.

[34] Kuznetsova A., Brockhoff P., Christensen R. (2016). Lmertest: Tests in Linear Mixed Effects Models. R Package.

[35] Lenth R. (2018). Estimated Marginal Means, aka Least-Squares Means. R Package.

[36] Brading M., Marsh P. (2003). The oral environment: The challenge for antimicrobials in oral care products. Int. Dent. J..

[37] Zuckerbraun H., Babich H., May R., Sinensky M. (1998). Triclosan: Cytotoxicity, mode of action, and induction of apoptosis in human gingival cells in vitro. Eur. J. Oral Sci..

[38] Jeyakumar J., Sculean A., Eick S. (2020). Anti-biofilm Activity of Oral Health-care Products Containing Chlorhexidine Digluconate and Citrox. Oral Health Prev. Dent..

[39] Carrouel F., Conte M.P., Fisher J., Goncalves L.S., Dussart C., Llodra J.C., Bourgeois D. (2020). COVID-19: A Recommendation to Examine the Effect of Mouthrinses with beta-Cyclodextrin Combined with Citrox in Preventing Infection and Progression. J. Clin. Med..

[40] Lo Giudice R. (2020). The Severe Acute Respiratory Syndrome Coronavirus-2 (SARS CoV-2) in Dentistry. Management of Biological Risk in Dental Practice. Int. J. Environ. Res. Public Health.

[41] Schlievert P., Peterson M. (2012). Glycerol monolaurate antibacterial activity in broth and biofilm cultures. PLoS ONE.

[42] Takahashi-Abbe S., Abbe K., Takahashi N., Tamazawa Y., Yamada T. (2001). Inhibitory effect of sorbitol on sugar metabolism of Streptococcus mutans in vitro and on acid production in dental plaque in vivo. Oral Microbiol. Immunol..

[43] Dutra-Correa M., Leite A., de Cara S., Diniz I., Marques M., Suffredini I., Fernandes M., Toma S., Araki K., Medeiros I. (2018). Antibacterial effects and cytotoxicity of an adhesive containing low concentration of silver nanoparticles. J. Dent..

[44] Hiraishi N., Yiu C., King N., Tay F., Pashley D. (2008). Chlorhexidine release and water sorption characteristics of chlorhexidine-incorporated hydrophobic/hydrophilic resins. Dent. Mater..

[45] Tüzüner T., Güçlü Z., Hurt A., Coleman N., Nicholson J. (2018). Release of antimicrobial compounds from a zinc oxide-chelate cement. J. Oral Sci..

[46] Wiedemann W., Lahrsow J., Naujoks R. (1979). The effect of periodontal resistance on experimental gingivitis. Dtsch. Zahnarztl. Z..

[47] Goes P., Dutra C., Lisboa M., Gondim D., Leitão R., Brito G., Rego R. (2016). Clinical efficacy of a 1% Matricaria chamomile L. mouthwash and 0.12% chlorhexidine for gingivitis control in patients undergoing orthodontic treatment with fixed appliances. J. Oral Sci..

[48] Takayama S., Kato T., Imamura K., Kita D., Ota K., Suzuki E., Sugito H., Saitoh E., Taniguchi M., Saito A. (2015). Effect of a mouthrinse containing rice peptide CL(14-25) on early dental plaque regrowth: A randomized crossover pilot study. BMC Res. Notes.

[49] Villa O., Ramberg P., Fukui H., Emilson C., Papanikolaou G., Heijl L., Birkhed D. (2018). Interaction between chlorhexidine and fluoride in a mouthrinse solution-a 4-day and 6-week randomized clinical pilot study. Clin. Oral Investig..

[50] Zimmer S., Kolbe C., Kaiser G., Krage T., Ommerborn M., Barthel C. (2006). Clinical efficacy of flossing versus use of antimicrobial rinses. J. Periodontol..

