# Supplementary material for: Toothpastes with Enzymes Support Gum Health and Reduce Plaque Formation
Source: Int J Environ Res Public Health. 2021 Jan 19;18(2):835. doi: 10.3390/ijerph18020835 (PMC7835853; doi:10.3390/ijerph18020835)
Supplement: Supplementary file 1 [file ijerph-18-00835-s001.zip › Supplemented Table S2.pdf]

Supplement Table S1

The base formulation of the enzyme-containing toothpastes served as placebo reference toothpaste in the laboratory testing (000) and in the clinical testing. The composition was based on the commercially available Enzycal 950 toothpaste (CURADEN, Kriens, Switzerland).

Ingredients	% W/W
Water	26.734
Sorbitol solution 70 % PhEur	28.5
Glycerin 1.23	15
Sident 8	12
Sident 22 S	8.077
Mulsifan CSA 20 (water soluble)	4
Veegum D	2
Hombitan AFDC 170 nm	1
Pepso 444348 (Luzi)	0.7
Sodium phosphate dibasic	0.5
Genuvisco TPC 1 (Carrageenan)	0.5
Sodium chloride	0.33
Sodium fluoride	0.229
Citric acid	0.2
Sodium benzoate	0.15
Sodium saccharin 450-fold	0.08
