# Supplementary figures and images for: Toothpastes with Enzymes Support Gum Health and Reduce Plaque Formation
Source: Int J Environ Res Public Health. 2021 Jan 19;18(2):835. doi: 10.3390/ijerph18020835 (PMC7835853; doi:10.3390/ijerph18020835)

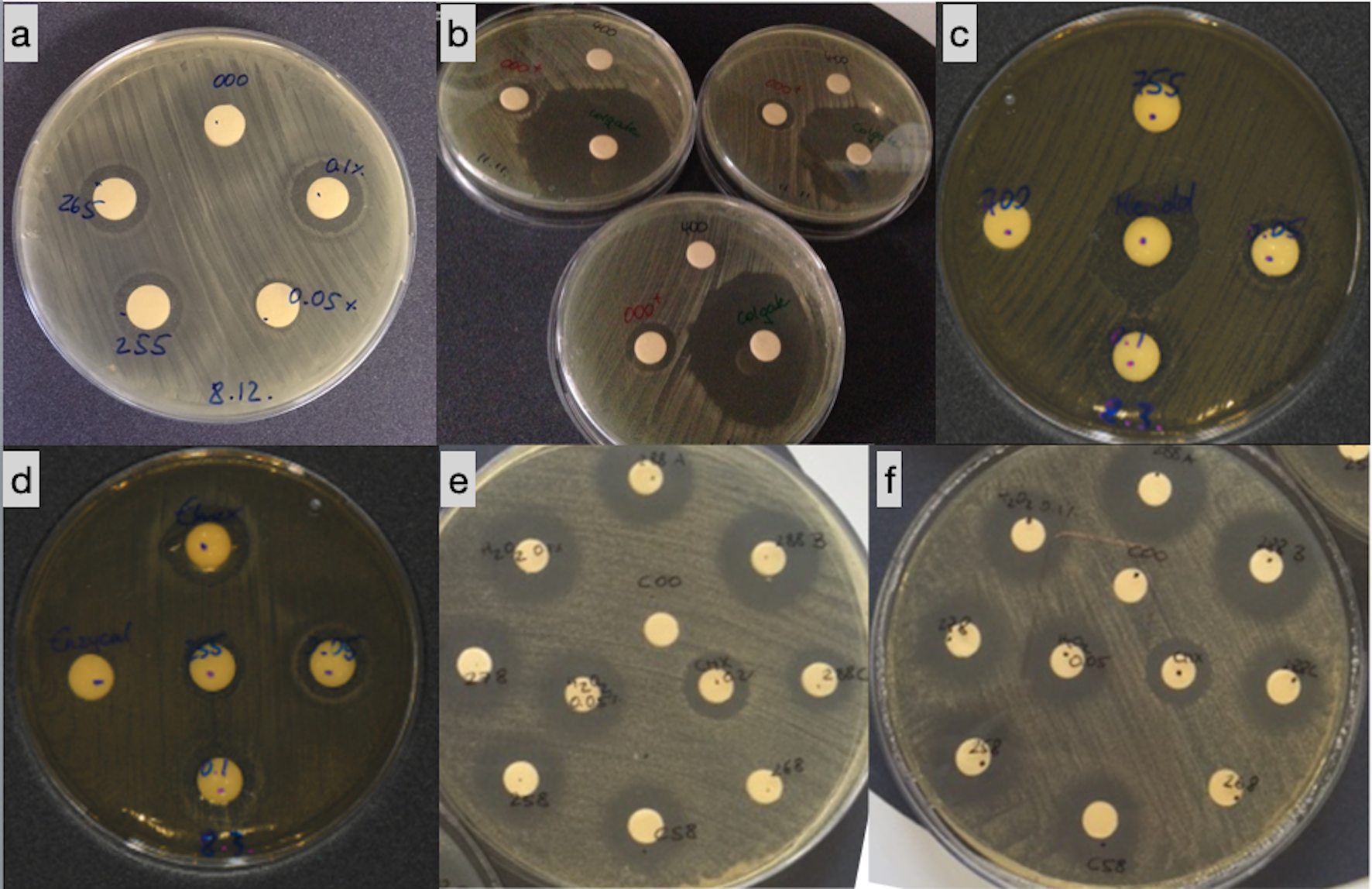

Supplement: Supplementary file 1 [file ijerph-18-00835-s001.zip › Supplemented Data S1.tiff]
